# Low foetal heart rate, a potentially ominous finding: case report

**DOI:** 10.1093/ehjcr/ytae440

**Published:** 2024-09-01

**Authors:** Andreea Sorina Afana, Cristina Filip, Brindusa Cimpoca, Ioana Dumitrascu-Biris, Ruxandra Jurcut

**Affiliations:** Expert Center for Genetic Cardiovascular Diseases, Emergency Institute for Cardiovascular Diseases, 258 Fundeni Street, Bucharest 022328, Romania; Neonatal Intensive Care Unit, University of Medicine and Pharmacy Carol Davila, 8 Eroii Sanitari Blv., Bucharest 050474, Romania; Department of Obstetrics and Gynecology, University of Medicine and Pharmacy Carol Davila, 8 Eroii Sanitari Blv., Bucharest 050474, Romania; Fetal Medicine Department, Filantropia Clinical Hospital, 11-13 Ion Mihalache Blv., Bucharest 011171, Romania; Department of Cardiothoracic Pathology, University of Medicine and Pharmacy Carol Davila, 8 Eroii Sanitari Blv., Bucharest 050474, Romania

**Keywords:** Congenital long QT syndrome, Long QT syndrome type 1, Foetal bradycardia, Potassium channels, Channelopathy, Case report

## Abstract

**Background:**

Congenital long QT syndrome (LQTS) type 1 is characterized by abnormally prolonged ventricular repolarization caused by inherited defects in cardiac potassium channels. Patients are predisposed to ventricular arrhythmias and even sudden cardiac death. In some cases, foetal sinus bradycardia is the only sign, making prenatal diagnosis challenging. Physicians should be aware of this subtle presentation of LQTS. Early diagnosis and proactive treatment are crucial for preventing unexpected cardiac events.

**Case summary:**

A healthy and asymptomatic 25-year-old pregnant woman was referred to our institute for cardiac evaluation after persistent foetal sinus bradycardia was detected during repeated ultrasounds, despite the absence of any foetal morphological or functional cardiac anomalies. After a thorough assessment, the mother was diagnosed with LQTS type 1, as confirmed by molecular genetic testing. Appropriate management, including maternal medication and increased surveillance, was initiated. The infant was delivered safely, and his electrocardiogram revealed a significantly prolonged QTc interval. Genetic testing confirmed the maternally inherited variant in KCNQ1 gene, and beta-blocker therapy was started. No arrhythmic events were noted.

**Discussion:**

Detection and careful stratification of foetal heart rate (FHR) is crucial in every pregnancy. Foetal bradycardia can be caused by both maternal and foetal factors. Persistent low FHR should raise a high suspicion for LQTS. The condition may also present with atrioventricular blocks, torsades de pointes, or sudden intrauterine foetal demise. Accurate and early diagnosis of LQTS is essential for implementing appropriate management strategies, which include vigilant monitoring, effective medical treatment, careful planning of delivery, and post-natal care.

**Learning points**

- In the majority of long QT syndrome type 1 patients, foetal sinus bradycardia, defined as foetal heart rate < 110 b.p.m. (or <3rd percentile for gestational age), is the only foetal arrhythmia detected *in utero* on routine prenatal ultrasound.
- Transplacental therapy generally consists of beta-blockers, most often in the form of propranolol because of its efficient transplacental passage.

## Introduction

In clinical practice, foetal echocardiography and cardiotocography have been the cornerstones for assessing foetal well-being and identifying foetal arrhythmias.

Foetal bradycardia is defined as a foetal heart rate (FHR) of less than 110 b.p.m. by the American College of Obstetrics and Gynecology^1^ and the International Society of Ultrasound in Obstetrics and Gynecology.^2^ Additionally, specific percentiles for gestational age are used in FHR monitoring, as rates below the 3rd percentile can accurately identify conduction disease.^3^ Foetal bradycardia can be attributed to various aetiologies, including maternal hypothyroidism, effects from maternal medications (such as beta-blockers and antithyroid agents), maternal antibody-induced sinus node dysfunction (anti-Ro/anti-La antibodies), congenital complete heart block, long QT syndrome (LQTS), and other inherited channelopathies. The differential diagnosis is important because it allows for the selection of the most effective therapeutic strategies.

Long QT syndrome is responsible for 15–17% of cases of foetal bradycardia with heart rates below 110 b.p.m. in foetuses with a normally structured heart.^4^

Diagnosing LQTS *in utero* is especially difficult and frequently delayed, as routine ultrasound findings are usually subtle, often revealing only incidental cases of sustained foetal bradycardia. Diagnosing LQTS before birth is crucial, as it is responsible for a significant number of late-term foetal losses and cases of sudden infant death.^5^

## Summary figure

**Figure:**
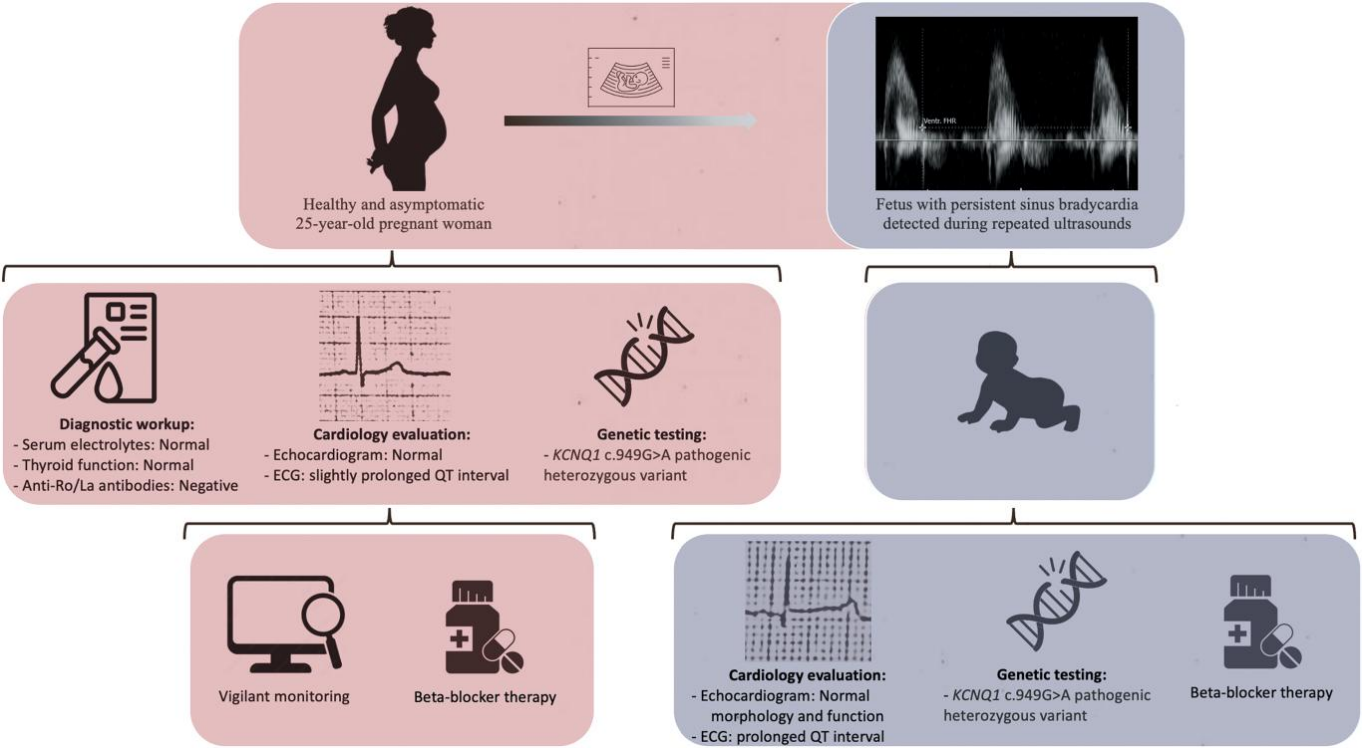
An apparently healthy pregnant woman was found to have a foetus with persistent sinus bradycardia. Comprehensive evaluation revealed LQTS type 1, leading to increased surveillance and beta-blocker therapy. Post-natal evaluation confirmed that the infant had a prolonged QTc interval, with genetic testing identifying a maternally inherited KCNQ1 variant. Beta-blocker therapy was initiated for the infant.

## Case presentation

A healthy 25-year-old woman, 32 weeks pregnant with good prenatal care, was referred to adult cardiology for evaluation after her foetus was found on routine foetal ultrasounds to have bradycardia. Her obstetric, personal, and family histories were otherwise unremarkable. This was her second pregnancy, following an uncomplicated first.

**Figure 1:**
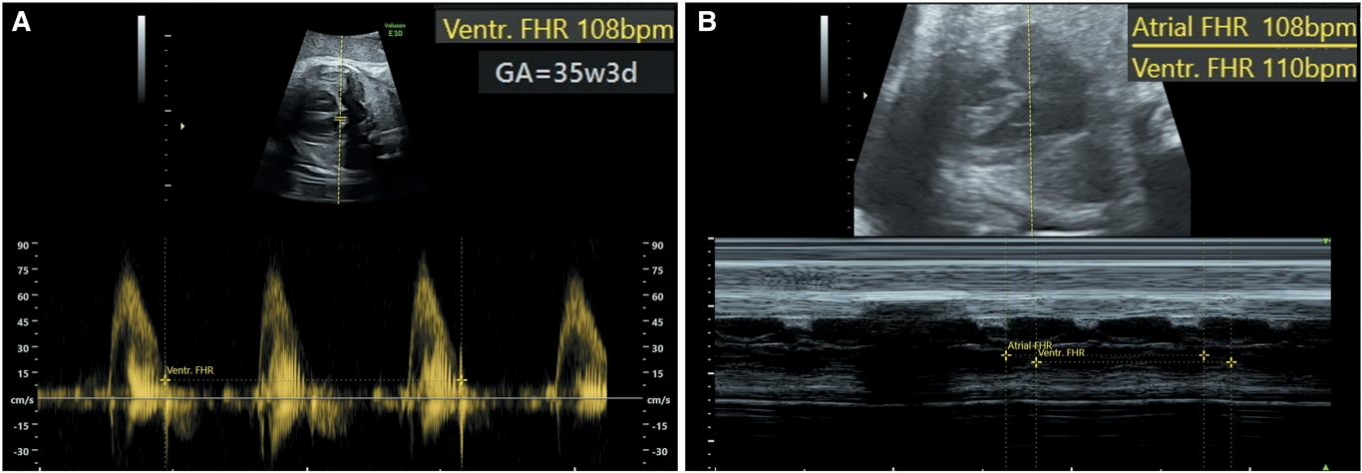
Foetal echocardiography at 35-week gestation showing a ventricular heart rate of 108 b.p.m. (*A*) with normal atrioventricular intervals (*B*).

Foetal bradycardia was initially detected in the first trimester. Her primary physician continued to closely monitor the foetal heart rate through serial ultrasounds. She was referred to a foetal medicine specialist for a more comprehensive evaluation, where the foetal echocardiogram revealed normal chamber dimensions and good ventricular function. On follow-up, the most recent examination at 35-week gestation showed no morphological or functional cardiac anomalies, but the sinus bradycardia persisted, with FHR ranging from 108 to 119 b.p.m. (*Figure 1*).

The mother was not undergoing any treatment with bradycardic effects. As part of an initial evaluation, laboratory testing for anti-Ro/La antibodies was performed, revealing negative status. Her thyroid status and serum electrolyte levels were normal.

**Figure 2:**
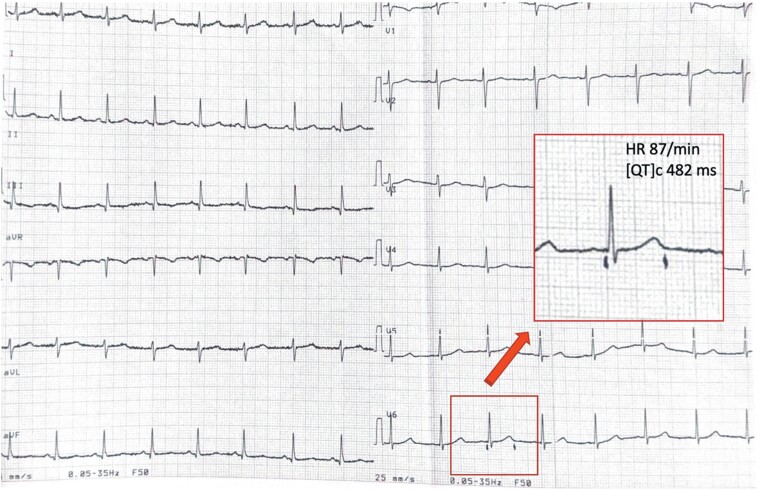
Electrocardiogram of the mother showing a slightly prolonged QTc interval of 482 ms.

The maternal echocardiogram was unremarkable, but her electrocardiogram (ECG) demonstrated a slightly prolonged QT interval (corrected QT interval 482 ms based on Bazett’s formula) (*Figure 2*). The paternal ECG was normal.

There was no maternal history or symptoms of arrhythmias. Genetic testing for LQTS, using a dedicated panel of 18 genes (Blueprint Genetics, Finland), was offered to the pregnant woman. The results revealed a pathogenic heterozygous variant (c.949G>A, p.Asp317Asn, NM_000218.3) in the *KCNQ1* gene, which encodes the alpha subunit of a voltage-gated K+ channel and is associated with autosomal dominant LQTS type 1. Despite being asymptomatic, the pregnant woman began treatment with beta-blockers (propranolol).

**Figure 3:**
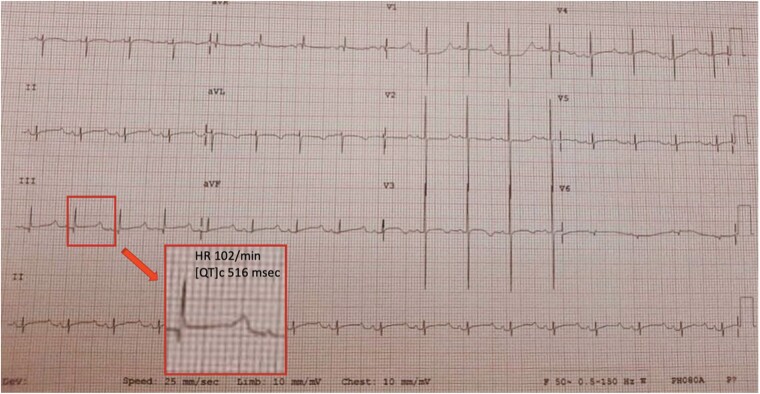
Electrocardiogram of the neonate showing sinus bradycardia with prolonged QT interval of 516 ms.

A caesarean delivery was planned owing to concerns about potentially inadequate cardiac monitoring during vaginal delivery. The infant was delivered at 37-week gestation with an APGAR score of 9/9 and remained haemodynamically stable the entire time. Post-natal echocardiogram showed bradycardic rhythm in the presence of otherwise normal findings. Electrocardiography confirmed the diagnosis of significant QT prolongation (corrected QT interval 510–561 ms) (*Figure 3*).

The neonate was started on propranolol to sustain the reduction in QT interval, with the dose gradually increased to a daily dosage of 2 mg/kg/day, under strict ECG monitoring due to the low heart rate. Post-natal genetic testing confirmed the final diagnosis of a pathogenic, maternally inherited variant in *KCNQ1* (c.949G>A, p.Asp317Asn). The infant was discharged home and continues to do well, with no arrhythmic events. Over the 10-month follow-up, the infant displayed normal growth and neurodevelopment and remained asymptomatic, but continued to have a slightly prolonged QTc interval (480–490 ms) despite being on propranolol prophylaxis. Genetic testing for LQTS was also recommended for the firstborn child, and the results were negative. Parents were educated about medications that should be avoided due to their potential to prolong QT intervals, and they were advised to treat promptly any intestinal disorders that can lead to low electrolytes level, to avoid intense physical activity and extreme emotional stress in the future.

## Discussion

Congenital LQTS is a group of inherited channelopathies, primarily involving three major autosomal dominant subtypes (types 1, 2, and 3), LQTS type 1 being the most common.^6,7^ These conditions are linked to impaired cardiac repolarization and an increased risk of life-threatening ventricular arrhythmias at any age. Foetuses with LQTS type 1 have a significantly shorter QTc interval compared with the other two types,^8^ and they generally have a more favourable prognosis.^9^

The prenatal diagnosis is particularly challenging and requires the recognition of specific rhythm abnormalities unique to the foetus. In the majority of LQTS type 1 patients, foetal sinus bradycardia is often the only foetal arrhythmia detected *in utero* on routine prenatal ultrasound.^3^ The post-natal diagnosis of LQTS is typically clear-cut, indicated by a prolonged QT interval revealed by an ECG, a positive family history, or characteristic LQTS arrhythmias. This diagnosis can be confirmed through genetic testing, as it was in the case of this child.

Ideally, LQTS should be diagnosed before birth to ensure optimal management for both the mother and the child. During foetal life, the most effective treatment is transplacental therapy, which generally consists of beta-blockers, most commonly in the form of propranolol, due to its efficient passage across the placenta.^10^ The treatment is typically tailored according to the dynamically changing status of both the mother and the foetus. Awareness of foetal LQTS will prompt the withholding or reduction of QT-prolonging maternal medications commonly used in obstetrics, such as oxytocin or ondansetron. An implantable cardioverter-defibrillator (ICD) is recommended post-natal for patients who do not respond to beta-blocker therapy and are at high risk for sudden cardiac death.^11^ Given that the infant responded well to the medical therapy and did not experience any arrhythmic events, the potential benefits of an ICD did not justify the associated risks. Recognition of maternal LQTS is equally important, as the risk of cardiac events increases during the postpartum period.^12^ Risk stratification for the expectant mother is a crucial step, as outlined in the 2018 ESC guidelines for managing cardiovascular diseases during pregnancy.^13^ This includes implementing varying levels of surveillance in specialized centres, choosing the appropriate method and location for delivery, and determining whether to start or stop specific medications.^14^ Such patients need close multidisciplinary follow-up for well-being and complications, delivery in a tertiary facility where comprehensive cardiac care is available for both the mother and the newborn.^15^

## Patient’s perspective

A patient coming from a family without any known cardiac diseases should receive adequate genetic counselling, and predictive family screening should be offered to all first-degree relatives. In LQTS patients, education should include avoiding drugs and situations which could further prolong QT interval. Our young mother and her spouse showed a good understanding of all medical explanations.

## Data Availability

The data underlying this article are available in the article and in its online supplementary material.

## Lead author biography

Dr Afana Andreea Sorina studied medicine at the University of Medicine and Pharmacy of Craiova, Romania. She is currently a cardiology resident specializing in multimodal cardiovascular imaging at the MIRACL.ai Laboratory, Multimodality Imaging for Research and Analysis Core Laboratory, and Artificial Intelligence at the University Hospital of Lariboisière (AP-HP) in Paris, France. Her research focuses on genetic cardiac disorders, and she undertook a 1.5-year training at the Expert Center for Rare Genetic Cardiovascular Diseases, Emergency Institute for Cardiovascular Diseases Prof. Dr C.C. Iliescu in Bucharest, Romania.
